# Effect of Powder from Different Jackfruit (*Artocarpus heterophyllus* Lam.) Sections on Performance, Blood Indices, Carcass Characteristics, and Meat Quality of Rabbits

**DOI:** 10.3390/ani15111609

**Published:** 2025-05-30

**Authors:** Liliana Ortega-González, Sergio Soto-Simental, Roberto González-Tenorio, Juan Ocampo-López, Héctor Hernández-Domínguez, Gerardo M. Nava-Morales, Maricela Ayala-Martínez

**Affiliations:** 1 Instituto de Ciencias Agropecuarias, Universidad Autónoma del Estado de Hidalgo, Av. Universidad s/n Km 1, Ex Hacienda de Aquetzalpa, Tulancingo CP. 43600, Hidalgo, Mexico; or231480@uaeh.edu.mx (L.O.-G.); sotos@uaeh.edu.mx (S.S.-S.); rtenorio@uaeh.edu.mx (R.G.-T.); jocampo@uaeh.edu.mx (J.O.-L.); 2 Bioterio, Circuito Ex Hacienda la Concepción s/n Carr. Pachuca-Actopan, San Agustín Tlaxiaca CP. 42160, Hidalgo, Mexico; hector_hernandez7859@uaeh.edu.mx; 3 Facultad de Química, Universidad Autónoma de Querétaro, Cerro de las Campanas S/N-Edificio 5, Santiago de Querétaro CP. 76017, Querétaro, Mexico; gerardomnava@gmail.com

**Keywords:** waste jackfruit, rabbit, meat quality

## Abstract

Fruit wastes have been considered as environmental pollution. Jackfruit is a good source of nutrients and bioactive compounds, meaning that its use in animal feed could contribute to waste reduction. The objective of this research was to evaluate the different parts of jackfruit (seed, pulp, and peel) as an agro-industrial waste on the productive parameters, carcass traits, and meat quality of rabbits. For this study, 144 thirty-five-day-old rabbits were randomly divided into four treatments, control (C), 2.5% pulp powder (PY), 2.5% seed powder (SY), and 2.5% peel powder (CY), with six repetitions and 6 rabbits for each repetition. The fattening period was 30 days. The best feed conversion ratios were found in the C, PY, and CY groups (*p* < 0.05). The highest chilled carcass yield was observed in the PY group (*p* < 0.05). Similar results were observed for intestinal morphology in all treatments (*p* > 0.05). The meat color obtained from the PY group had higher whiteness, redness, and chroma values (*p* < 0.05). Higher taste and general acceptability values for meat were observed in the groups using jackfruit (SY, PY, and CY). It is concluded that the addition of jackfruit peel powders can be used in rabbit feed, as they improve the final weight, feed conversion, carcass yield, and meat texture, while the sensory analysis demonstrates that the meat is well accepted by consumers.

## Simple Summary

Jackfruit parts are considered a good source of nutrients, so their use in animal feed could contribute to waste reduction. In this study, jackfruits were divided into seed, pulp, and peel, and the parts were used to feed rabbits. After four weeks, the animals were slaughtered, and carcass traits and meat characteristics were evaluated. Then, burgers were made from the meat obtained. The results found in this study indicate that pulp and peel powders have a feed conversion ratio similar to the control group of rabbits. However, a sensory analysis specified that good taste and general acceptability were observed in the groups using jackfruit parts. According to these results, the addition of jackfruit parts to feed rabbits can be used to fatten rabbits and contribute to obtaining meat with high consumer acceptability.

## 1. Introduction

In Mexico, there are three main types of rabbit farmers, namely small, medium, and business farmers, with 50% of all rabbit farmers being medium-scale producers; these rabbit farmers use commercial feed and sometimes add a local plant [1]. Over the past few years, commercial rabbit feed has shown considerable noncompliance with the nutrient levels indicated by the manufacturer; specifically, it does not comply with the minimum recommended levels of crude fiber and crude protein [2]. In addition, the human population has grown significantly worldwide, and, therefore, there is a great demand for meat to satisfy the food needs of this expanding population. This has encouraged animal breeders to identify alternative sources to ensure the formulation of diets for their animals at a lower cost [3] and provide a better supply of nutrients.

It has been proven that a wide range of fruits rich in different nutrients is being threatened by the lack of demand for fresh produce and crop damage suffered during the rainy season [4]. It is for these reasons that the utilization of agro-industrial waste (fruit wastes, agricultural pulp wastes, crop residues, sun-dried brewers’ grains, and the pomace of some fruits) in animal feed has been proposed as a promising alternative for meat production. Also, it contributes to sustainable agriculture as well as improving meat quality [5]. The feasibility of using fruit and vegetable residues for feeding different animals has been proven [6].

Jackfruit (*Artocarpus heterophyllus* Lam.) is considered one of the main indigenous fruits of India [4]. This fruit is cultivated in subtropical zones, including in countries in Asia, Africa, and the Americas, where yields up to 26 ton·ha**^−^**^1^ have been obtained [7]. Likewise, it has an abundance of essential amino acids, minerals, vitamin C, and bioactive compounds that give it antioxidant, anti-inflammatory, anticancer, antidiabetic, and antiviral properties [8]. The utilization of residues from this fruit as from many others contributes to the reduction in the impact of waste discharged into the environment [9]. Different researchers have evaluated the addition of this fruit in the feed of different animals, such as tilapia [10], goats [11], broilers [12,13], and West African Dwarf Bucks [14].

Rabbit farming is an activity that focuses on raising rabbits to obtain white meat, which is considered beneficial for the human body due to its supply of essential fatty acids, proteins, vitamins, and minerals [15]. For this animal species, some agro-industrial residues and coproducts have been used [9]. It would be expected that jackfruit could be a good source of nutrients and bioactive compounds to be used in feed for fattening rabbits.

The objective of this research was to evaluate the effects of the use of different parts of jackfruit (seed, pulp, and peel) as an agro-industrial waste feed additive on the productive parameters, carcass quality, and meat quality of rabbits.

## 2. Materials and Methods

This study was approved by the institutional committee for the care and use of laboratory animals under act number CICUAL-V-I/011/2023.

### 2.1. Jackfruit Flour

Mature jackfruit was obtained from Xicotepec de Juarez, Puebla. The fruit was collected from a fruit orchard located in a subtropical zone at an altitude of 1100 m above sea level. Then, the fruit was transported to the lab, where it was washed, cut, and divided into separate sections (seed, pulp, and peel) that were dried at 60 °C for 72 h [16] using a Riossa HCF-82 dryer (TMP Equipos, Mexico City, Mexico).

### 2.2. Animals and Treatments

A total of 144 California X New Zealand crossbred rabbits with an age of 35 days and average weight of (1019.62 ± 140.15 g) were randomly divided into one of the following four groups: C (Control), SY (2.5% jackfruit seeds), PY (2.5% jackfruit pulp), and CY (2.5% jackfruit peel). Each group contained six repetitions with six rabbits. All diets (Table 1) were isoproteic (16%), isoenergetic (2.5 Mcal·kg DM^−1^) and isofibrous (NDF 30% and ADF 17%) according to the nutritional requirements for rabbits [17], while the nutritional composition of the ingredients was obtained from the Fundación Española para el Desarrollo de la Nutrición Animal [18]. All ingredients were blended using an ASF MZ50 double helicoidal mixer (Molinos y Mezcladoras Industriales S. A. de C. V., Mexico City, Mexico), and subsequent pellets were obtained using a SKJ120 pelletizer (Yuezhen Machinery Co., Jinan, China). All animals were provided feed and water ad libitum for 28 days. The rabbits from each repetition were housed in a space measuring 45 × 60 × 40 cm, which was adapted with automatic drinkers and manual feeders, and an ambient temperature and humidity of 21.5 °C and 43.1%, respectively. The feeding of the rabbits began every day at 8:00 am. Uneaten feed was weighed, and then a quantity of feed weighing 50 g per rabbit was offered, which was then increased to 200 g per day by the end of the fattening period.

All rabbits were slaughtered at the age of 65 days according to the national legislation for the slaughtering of animals [19].

### 2.3. Productive Performance Parameters

The feed consumption was measured daily with the rejected and offered feed weighed, while body weight was measured weekly using a Mettria MTNUV-40 scale (Mettria, Mexico City, Mexico). The collected data were used to calculate the average daily gain, average feed intake, and feed conversation ratio.

### 2.4. Blood Collection

At the time of slaughtering, blood samples (2 mL per animal) were collected from 6 animals per treatment in a sterile tube with ethylenediaminetetraacetic acid and sent to the laboratory for analysis to determine blood biometry using a Exigo-H400 hematology analyzer (Kabla Veterinary DX, Mexico City, Mexico). Another sterile tube without an anticoagulant was used to obtain serum to quantify biochemical compounds with a BK-1200 biochemistry analyzer (BioBase Biodusty, Jinan, China).

### 2.5. Small Intestine Histology

Samples (5 cm) from six rabbits per treatment were obtained from the middle sections of the small intestine (duodenum, jejum, and ileum) of the rabbits fed with four treatments (C, SY, PY, and CY). Each section was processed using the paraffin-embedding method [20] using a Microm model TP120 automatic tissue processor (Thermo Fisher Scientific, Walldorf, Germany). Subsequently, the samples were stained using the hematoxylin and eosin method in order to cover the samples with a synthetic resin [20]. Lastly, the samples were analyzed using a brightfield Olympus BX41 microscope (Olympus Corporation, Tokyo, Japan). The tissue images of the small intestine were captured using ImagePro ver. 6.0 software.

### 2.6. Carcass Traits

Carcasses were evaluated according to the recommendations described by Blasco et al. [21] with the following modifications: the cuts made were to obtain the head (cut point between occiput and atlas vertebra), fore part (section between the atlas vertebra 6th thoracic vertebra), intermediate part (section between the 7th and last thoracic vertebra), hind part (section between the 1st and 7th lumbar vertebra), and complete hind legs (without the muscle insertion of the hind legs). The complete viscera (including digestive system, liver, bladder, heart, kidneys, reproductive apparatus, and spleen) were weighed in their entirety and separately from the heart, liver, and kidneys. All measurements were carried out using a Torrey L-PCR scale (Torrey, Monterrey, Mexico). The percentage yield of all the sections was calculated.

### 2.7. Meat Characteristics

All carcasses (*n* = 30 rabbits by treatment) obtained from the slaughtered rabbits were used to determine meat characteristics. The meat quality was evaluated after cooling for 24 h at 8 °C. The pH levels of the *Longissimus lumborum* muscles were measured using a Hanna HI99163 pH-meter (Hanna Instruments, Cluj-Napoca, Romania). Meat color was determined using the color space of CIEL*a*b* with a LS171 Linshang colorimeter (Shenzhen Lingshang Technology Co., Shenzhen, China) following the indications described by King et al. [22]. Water-holding capacity (WHC) was evaluated according to the methodology described by Honikel et al. [23]. Cooking losses were measures in the loins, with the samples placed in a plastic bag and then cooked al 80 °C. Subsequently, they were cooled to room temperature, and, finally, the calculation was determined by weight differences in percentage. The texture profile analysis (TPA) was evaluated on the cold meat; the samples were cut into cubes of 1 cm on each side, and a 50% compression was used perpendicular to the direction of the muscle fiber using a 1 mm·s^−1^ velocity, a 3.5 cm diameter aluminum probe, and a standard base. Once the test was performed, the parameters of hardness, resilience, cohesiveness, elasticity, and chewiness were obtained according to the indications of Bourne [24] using Exponent ver. 6.2.4.0, which controls the texture analyzer model TA-X-T PLUS (Stable Micro Systems, Surrey, UK).

### 2.8. Sensorial Analysis

The meat obtained from the legs (3 kg per treatment) of the four groups (C, SY, PY, and CY) was evaluated by means of an affective hedonic test, which was performed to determine acceptability levels. Eighty consumers with an average of age of twenty-three years were recruited to evaluate the samples, of which 57.5% were female and 42.5% were male. The burgers were wrapped in aluminum foil and then cooked for approximately two minutes using a grill (Vollart, Mexico City, Mexico) until reaching a temperature of 68 °C. The samples were cut into four pieces, and one from each treatment was given to the panelists on a disposable plate. Each meat burger piece was identified using a random three-digit number. The attributes evaluated for the cooked meat were as follows: odor, firmness, juiciness, taste, and general acceptability, as well as raw meat color (white, red, and yellow). The tests were carried out in sensorial analysis laboratory booths, which were complied with international requirements as indicated in the ISO 8589 standard [25].

### 2.9. Statistical Analysis

Data was analyzed by ANOVA using a general linear model (carcass traits, meat characteristics, and sensory analysis) and mixed model (feed consumption, daily weight gain, weekly weight, and the feed conversion ratio) using the following equations:

Yij = μ + αi + εij

Yij = μ + αi + βj + αi(βj) + εij

where Yij = response variable, µ = population media general, αi = Factor A (experimental diets), βj = Factor B (weeks), αi(βj) = nested effect, and εij = experimental error

This study was performed by completely random design. The differences between the averages were evaluated using LSMEANS option (*p* < 0.05). All data were analyzed using SAS software version 9.0.

## 3. Results

### 3.1. Productive Performance Parameters

The results of the productive parameters are presented in Table 2. It can be observed that during the first week, the animals from the SY group consumed the least amount of feed, while during the second week, the animals from the SY and PY groups consumed the least (*p* < 0.05). The highest weight gain during the first week was in rabbits from groups C, PY, and CY, while during the fourth week, it was in groups C and SY (*p* < 0.05). The highest weekly weight (*p* < 0.05) during the second and third weeks was found in groups C, PY, and CY. However, at the end of fattening, the CY group reported the highest average weekly weight. Feed conversion in the second and fourth weeks was better in the C and CY groups (*p* < 0.05).

### 3.2. Blood Evaluation

When analyzing the blood biochemical parameters of the rabbits in this research (Table 3), the levels of blood urea nitrogen and creatinine were higher (*p* < 0.05) in the rabbits that consumed some jackfruit sections (SY, PY, and CY).

As for total alkaline phosphatase, all groups presented values higher than expected for the species; however, the animals that consumed some sections of the jackfruit (SY, PY, and CY) reported lower values compared to group C (*p* < 0.05).

### 3.3. Small Intestine Histology

**Figure 1:**
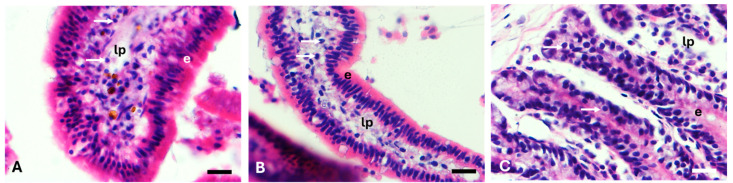
Small intestine of rabbits fed with jackfruit using different components of the fruit (*Artocarpus heterophyllus* Lam): (**A**) duodenum of PY treatment; (**B**) jejunum of PY treatment; and (**C**) ileum of SY treatment. Slash = 20 µm. Arrows = lymphocytic infiltration. lp = lamina propria. e = epithelium.

The evaluated sections of the rabbits’ small intestine (duodenum, jejunum, and ileum) were similar (*p* > 0.05) for the four groups during feeding with the different sections of the jackfruit. Representative images of the three evaluated sections can be seen in Figure 1. Epithelium, lamina propria, and lymphocytic infiltration were similar among the treatments.

### 3.4. Carcass Quality

Table 4 shows the results of the evaluation of carcass characteristics, where it can be observed that the PY group presented the highest percentage of yield for the cold carcass (*p* < 0.05). Moreover, groups C, PY, and CY reported a higher skin percentage (*p* < 0.05). When evaluating the whole viscera, groups SY, PY, and CY presented the highest percentage compared to group C. However, when evaluated individually, groups C, SY, and PY presented the largest livers, while group SY recorded the largest hearts and kidneys (*p* < 0.05). In the results for the evaluation of the primary cuts of the carcass, the groups containing animals that consumed a section of the jackfruit (SY, PY, and CY) presented a lower scapular fat percentage, larger legs, and a higher quantity of meat in this same cut compared to the control group (*p* < 0.05).

### 3.5. Meat Quality

The results for the evaluation of the rabbits’ meat color are presented in Table 5, where higher lightness (L*) values were found in groups C and PY (*p* < 0.05). In addition, a higher red index (a*) and higher color saturation (chroma) were found in groups SY and PY (*p* < 0.05), while all groups presented similar hue values (*p* > 0.05). In contrast, the lowest pH was recorded in groups C and SY (*p* < 0.05). Finally, the highest water-holding capacity (WHC) was found in group C (*p* < 0.05).

Table 6 shows that group SY presented the lowest cooking loss (*p* < 0.05), while for the texture profile analysis, the PY and CY groups reflected the lowest hardness and chewiness (*p* < 0.05).

### 3.6. Sensorial Analysis

For the sensory evaluation of rabbit meat (Table 7), consumers indicated that meat from groups C, SY, and PY had a greater odor intensity (*p* < 0.05). Groups C, SY, and CY presented greater hardness (*p* < 0.05), although all the meat treatments presented the same juiciness (*p* > 0.05). The addition of the different jackfruit sections improved the flavor and general acceptability of the meat compared to the control group (*p* < 0.05). Finally, when evaluating the intensity of the colors in the raw meat, it was observed that groups C and PY presented a greater intensity of white, CY a greater intensity of red, and C, SY, and PY a greater intensity of yellow (*p* < 0.05).

## 4. Discussion

Rabbit meat production plays an important role in ensuring an adequate supply of sustainable meat around the world [15]. Minimal initial investment is required to breed these animals, and their management is fairly straightforward. Therefore, optimizing the nutritional aspects would contribute to increasing their productivity [26]. In general, the groups fed with jackfruit sections, especially PY and CY, increased productive performance compared to control group. The feed consumption of the rabbits fed with jackfruit peel (CY) was low consumption but high body weight, indicating that the assimilation of the feed components is possibly better in this diet. To the best of our knowledge, there is little information about the use of jackfruit sections influencing carcass traits. In one study, however, the use of jackfruit leaves to feed goats increased productivity, including feed efficiency and body weight [27]. Muthukumar et al. [28] reported that the use of jackfruit waste in dairy cows increased milk production and quality. In a further investigation, it was concluded that the use of processed jackfruit seed can be used to increase weight gain in Nile tilapia [29]. The use other types of agro-industrial waste can affect the productivity of rabbits, as reported by Tavares et al. [30], who evaluated acerola in the diet of growing rabbits and described improved weight and feed intake. Menchetti et al. [26] incorporated goji berries into rabbit feed and obtained enhanced feed conversion and growth rates. It has been evidenced that carefully incorporating agro-industrial residues into rabbit diets can contribute to improved growth rates, feed conversion, and overall performance due to the richness of nutrients they provide [5]. In this research, jackfruit could have contributed to animal nutrition, as the presence of antioxidants, minerals, essential amino acids has been confirmed [31].

Blood analysis is an essential procedure for evaluating animal health [32]. According to Brandão et al. [33], all the values from this study are within the normal values reported for the species. High values of total alkaline phosphatase indicate the presence of a type of abnormal organic function. It is clear that the jackfruit could have contributed to reducing the risk of developing a pathology, although further studies would be needed to determine the true origin of these high values since this enzyme can be found in different areas of the body, such as the liver, bone, kidneys, and intestines [34]. However, it has been reported that processed jackfruit seed can induce low red blood cell counts in fish [29]. Jackfruit sections maintain animal health, leading to improved animal productivity. The use of jackfruit leaves to feed goats does not have an effect on blood biochemistry parameters [27]. Moreover, in other investigations involving the incorporation of agricultural waste such as passion fruit seed [35], acerola [30], and herbal mixtures [36] to the diet of rabbits, no alterations in the blood parameters were observed.

The structure and variety of cells in the small intestine create a complex environment, where digestion is facilitated by the absorption of nutrients [37]. The efficiency of jackfruit sections in productive performance could be associated with intestinal epithelial cell integrity, since Fitrya et al. [38] demonstrated that an ethanolic extract of jackfruit is effective in lowering the presence of peptic ulcers. Similarly, other studies have shown that the addition of agro-industry waste, such as pomegranate extract in rabbit feed, provided evidence of an apparent positive effect on the histological structure of the animals’ small intestine [39]. It has been shown that jackfruit peel presents polysaccharides, which promote the growth of beneficial flora when degraded in the intestine [40].

Carcass traits are influenced by the feed and its components, as well as the increase in feed consumption and body weight. However, raw jackfruit seed meal fed to Guinea fowl keets had no effect on cut parts and internal organs [41]. However, the use of agro-industrial waste or other plants in rabbits modified carcass traits, such as with the research described by Volek et al. [42], who evaluated white lupine seed in rabbit diets, reporting higher cold carcass yield and greater weight in the posterior paste from the carcass. In addition, the study suggested that the rapidly degradable proteins and the energy provided by lupine are efficiently utilized for the synthesis of tissues such as muscles, which in turn contributes to the higher weight achieved. Similarly, this phenomenon could have occurred in this research, since it has been proven that jackfruit provides proteins and essential amino acids [43], with the animals in group CY obtaining a higher body weight.

Meat color is an indicator of quality, as it is associated with a pleasing appearance by the consumer. The color of rabbit muscles is pale pink, although natural pigments present in fruits have been found to contribute to the intensification of redness and yellowness of the flesh [44]. It has been stated that jackfruit has different contents of natural pigments such as beta-carotene all-trans and lutein all-trans, which may have contributed to the intensification of the color. Rabbits that consumed jackfruit tended to have lower L* values and higher chroma values, which is related to higher feed consumption. This indicates that jackfruit sections contain molecules that provide color, as mentioned above.

During the rigor mortis process, hardening and acidification occur due to glycogenolysis, which are changes that influence meat quality. According to Menchetti et al. [45], the pH of the meat may have been influenced by the jackfruit, which affected glycogen storage and enzyme activity in the muscle. In one study that evaluated meat from rabbits fed with different residues such as passion fruit seed [35] and acerola [30], the researchers found pH values lower than those from this research.

The water-holding capacity of meat is an important indicator that determines visual acceptability, yield, and sensory traits at the time of consumption. The water lost during cooking is probably due to heat-induced protein denaturation during this process, which results in less water being trapped within the protein structures held by capillary forces [46]. The animals in the groups that consumed jackfruit sections had the lowest WHC values, indicating that the meat from the animals fed with this waste would be able to lose moisture. There are some factors, such as pH, ion availability, and degree of the myofibrillar proteins, that affect WHC. The jackfruit sections seem to modify the pH of the meat, which makes it possible to increase the ions available for water trapping. Similar results were found by Sosnówka-Czajka et al. [47], who fed dried fruit pomace to broilers. However, the use of goji berries to supplement the diet of fattening rabbits did not affect the WHC [26]. In another study, color, which is related to pH and WHC, was influenced by the use of citrus for feeding rabbits [48]. Lower cooking loss results in better meat quality, because during cooking, nutrient loss may occur [49]. However, in this study, cooking loss parameters were similar among the C, SY, and CY groups, which is related to the WHC of these groups. The cooking method employed for rabbit meat has an influence on WHC and cooking loss [50].

Meat texture is a multidimensional property describing structural, mechanical, and surface properties, which are all directly related to sensory appreciation by the consumer [51]. In works evaluating brown algae in rabbits, an improvement in meat texture and flavor has been reported, while the addition of polyphenol-rich sources probably protects proteolytic enzymes (calpain and m-calpain) from the oxidative process, increasing their functionality and consequently the tenderness of the meat [52]. In contrast to the results in this study, other works have reported that the use of herbs or other vegetal compounds do not have an effect on TPA parameters [53]. However, it is possible that some bioactive compounds present in jackfruit sections have an effect on meat hardness by modifying the action of the endogenous meat enzymes.

In a sensory analysis of rabbit meat carried out by Tavares et al. [30], it was stated that the use of substances with abundant phenolic compounds can provide greater integrity of the myofibrillar membranes and consequently an improvement in the texture of the meat. On the other hand, Kuang et al. [54] mentioned that a low fat content in muscle will lead to a loss of qualities such as texture and flavor. The addition of different plant sources resulted in the improvement of meat texture, juiciness, flavor, and acceptability, such as wine grape pomace [55], tomato pomace [56], plant extracts [57], and *Saccharina latissima* and *Himanthalia elongata* [58].

## 5. Conclusions

This research demonstrated that the addition of jackfruit pulp or peel can be used in rabbit feed with a 2.5% supplementation due to its multiple benefits. It was shown to improve the final weight, feed conversion, carcass yield, and meat texture, while the sensory evaluation showed that the meat obtained better consumer acceptance. These results provide valuable information for rabbit breeders and commercial feed manufacturers, which could potentially contribute to an increase in production and an improvement in meat quality.

## Figures and Tables

**Table 1:** Diets with different jackfruit (*Artocarpus heterophyllus* Lam.) sections added to feed rabbits.

Ingredients (g/Kg)	Treatments ^1^			
	C	SY	PY	CY
Barley	46.1	46.2	46.2	46.2
Corn	142.5	142.8	142.7	142.7
Sorghum	94.3	94.5	94.5	94.5
Dried distilled grains	89.3	71.6	71.6	89.4
Wheat bran	131.4	131.7	131.7	131.7
Cane molasses	101.7	101.9	101.9	101.9
Canola meal	46.4	46.5	46.5	46.5
Soybean meal	102.7	102.9	102.9	102.9
Soybean hull	139.4	139.7	139.7	139.6
Straw	80.8	72.0	72.0	54.0
Vitamins and minerals premix	25.0	25.0	25.0	25.0
Jackfruit seed	-	25.0	-	-
Jackfruit pulp	-	-	25.0	-
Jackfruit peel	-	-	-	25.0
Calculated nutrient	Protein 16%			
	Neutral Detergent Fiber 30%			
	Acid Detergent Fiber 17%			
	Metabolic Energy 2.5 Mcal·kg DM^−1^			

^1^ C = Control, SY = 5% jackfruit seed, PY = 5% jackfruit pulp, CY = 1% jackfruit peel.

**Table 2:** Least squares mean of productive performance of rabbits fed with different jackfruit (*Artocarpus heterophyllus* Lam.) sections.

Variable	Weeks	Treatments ^1^				
		C	SY	PY	CY	SEM ^2^
Feed consumption	1	105.71 ^bAB^	100.19 ^dB^	112.57 ^cA^	109.00 ^cA^	2.53
	2	139.18 ^aA^	129.26 ^cBC^	124.44 ^bC^	133.63 ^bAB^	2.53
	3	144.77 ^a^	141.97 ^b^	145.09 ^a^	142.00 ^a^	2.53
	4	144.73 ^a^	150.36 ^a^	147.35 ^a^	141.34 ^a^	2.36
	SEM ^2^	2.48	2.48	2.48	2.48	
Weight gain	1	45.53 ^aAB^	40.96 ^abB^	47.77 ^aA^	46.90 ^aA^	2.10
	2	41.11 ^ab^	35.90 ^b^	39.80 ^b^	41.72 ^a^	2.10
	3	45.45 ^a^	44.59 ^a^	44.45 ^ab^	45.52 ^a^	2.10
	4	36.57 ^bAB^	41.08 ^abA^	32.97 ^cB^	34.16 ^bB^	2.10
	SEM ^2^	2.10	2.10	2.10	2.10	
Weekly weight	Initial weight	1013.57 ^e^	1010.83 ^e^	1031.94 ^e^	1025.83 ^e^	28.38
	1	1328.75 ^d^	1297.58 ^d^	1366.38 ^d^	1354.16 ^d^	28.28
	2	1616.52 ^cAB^	1548.88 ^cB^	1645.00 ^cA^	1646.25 ^cA^	28.28
	3	1946.66 ^bAB^	1893.50 ^bB^	1974.00 ^bAB^	1984.50 ^bA^	30.98
	Final weight	2202.66 ^a^	2181.10 ^a^	2206.72 ^a^	2223.66 ^a^	31.12
	SEM ^2^	29.77	29.36	29.47	29.36	
Feed conversion ratio	1	2.34 ^c^	2.45 ^c^	2.37 ^c^	2.40 ^c^	0.16
	2	3.41 ^bAB^	3.61 ^bA^	3.15 ^bB^	3.22 ^bAB^	0.16
	3	1.88 ^c^	1.87 ^d^	1.96 ^c^	1.79 ^d^	0.16
	4	4.40 ^aB^	4.15 ^aB^	5.22 ^aA^	4.59 ^aB^	0.16
	SEM ^2^	0.16	0.16	0.16	0.16	

^1^ C = control, SY= 2.5% jackfruit seeds, PY = 2.5% jackfruit pulp, CY = 2.5% jackfruit peel. ^2^ SEM: Standard Error Media. ^abcde^: Different superscript lowercase letters among rows indicate statistical differences (*p* < 0.05). ^ABC^: Different superscript capital letters among columns indicate significant differences (*p* < 0.05).

**Table 3:** Least squares mean of complete blood count of rabbits fed with different jackfruit (*Artocarpus heterophyllus* Lam.) sections.

Variables	Treatments ^1^				SEM ^2^
	C	SY	PY	CY	
Blood count					
Total white blood cells × 10^12^·L^−1^	4.01	4.87	3.33	4.65	1.21
Total red blood cells × 10^12^·L^−1^	5.01	5.89	5.01	4.83	0.65
Hemoglobin g·L^−1^	112.50	133.20	111.83	107.20	1.41
Hematocrit (%)	35.52	42.40	36.27	34.45	4.67
Mean corpuscular volume (fL.)	71.20	71.42	72.32	71.37	1.12
Mean corpuscular hemoglobin (pg.)	22.60	22.38	22.38	22.30	0.42
Mean corpuscular hemoglobin concentration g·L^−1^	317.33	312.33	309.66	312.16	0.33
Platelet count × 10^9^·L^−1^	210.20	205.70	180.30	310.50	53.35
Differential leukocyte count					
Granulocytes (%)	45.22	49.22	53.92	51.30	4.10
Lymphocytes (%)	47.00	43.15	39.15	42.80	3.73
Monocytes (%)	7.78	7.45	6.93	14.00	4.24
Blood chemistry					
Glucose (mg·dL^−1^)	72.33	65.83	65.83	73.67	2.97
Urea (mg·dL^−1^)	27.08	32.78	34.18	33.65	2.12
Blood urea nitrogen (mg·dL^−1^)	12.66 ^b^	15.36 ^ab^	16.00 ^ab^	16.00 ^a^	0.99
Creatinine (mg·dL^−1^)	0.73 ^b^	1.13 ^a^	1.29 ^a^	1.10 ^a^	0.09
Uric acid (mg·dL^−1^)	0.20	0.39	0.33	0.36	0.10
Total cholesterol (mg·dL^−1^)	95.50	103.3	110.50	97.80	10.30
Triglycerides (mg·dL^−1^)	80.3	96.7	94.80	95.00	10.85
Total bilirubin (mg·dL^−1^)	0.51	0.37	0.63	0.49	0.07
Direct bilirubin (mg·dL^−1^)	0.20	0.17	0.25	0.20	0.04
Aspartate aminotransferase (U/L)	54.53	36.58	47.87	52.63	5.33
Alanine aminotransferase (U/L)	52.47	32.17	48.72	45.90	6.19
Total proteins (g/dL)	6.38	6.74	6.64	6.69	0.17
Albumin (g·dL^−1)^	3.95	4.12	4.57	3.88	0.26
Globulins (g·dL^−1^)	2.42	2.28	2.07	2.80	0.31
Total alkaline phosphatase (U/L)	298.50 ^a^	220.00 ^b^	199.70 ^b^	259.50 ^ab^	19.55

^1^ C = control, SY= 2.5% jackfruit seeds, PY = 2.5% jackfruit pulp, CY = 2.5% jackfruit peel. ^2^ SEM: Standard Error Media. ^ab^: Different superscript lowercase letters among columns indicate statistical differences (*p* < 0.05).

**Table 4:** Least squares mean of carcass characteristics from rabbits fed with different jackfruit (*Artocarpus heterophyllus* Lam.) sections.

Variable	Treatments ^1^				SME ^2^
	C	SY	PY	CY	
Back length (cm)	33.14	32.47	32.15	32.00	0.03
Lumbar girth (cm)	20.47 ^b^	20.12 ^b^	22.34 ^a^	19.03 ^b^	0.49
Hot carcass yield (%)	53.89	53.71	55.075	54.75	0.40
Chilled carcass yield (%)	52.07 ^c^	52.59 ^bc^	54.46 ^a^	53.41 ^b^	0.33
Carcass back length (cm)	32.93	32.43	32.00	30.54	0.75
Carcass lumbar girth (cm)	15.79	15.29	16.41	16.07	0.45
Skin (g·kg live weight)	14.49 ^a^	13.40 ^b^	14.30 ^a^	14.25 ^a^	0.18
Viscera (g·kg live weight)	21.08 ^b^	24.53 ^a^	23.45 ^a^	22.67 ^ab^	0.58
Liver (g·kg live weight)	3.70 ^ab^	4.10 ^a^	4.00 ^ab^	3.42 ^b^	0.15
Heart (g·kg live weight)	0.33 ^b^	0.48 ^a^	0.31 ^b^	0.32 ^b^	0.02
Kidneys (g·kg live weight)	0.68 ^b^	0.81 ^a^	0.63 ^b^	0.67 ^b^	0.02
Feet (g·kg live weight)	2.41	2.34	2.34	2.42	0.03
Drip losses (%)	3.37	2.32	1.98	2.81	0.37
Head (g·kg cold carcass weight)	9.77	9.73	9.35	12.28	1.58
Forepart (g·kg cold carcass weight)	24.11	24.69	24.65	24.70	0.16
Intermedia part (g·kg cold carcass weight)	10.20 ^ab^	10.88 ^a^	9.96 ^ab^	9.60 ^b^	0.29
Hind part (g·kg cold carcass weight)	20.93	28.76	20.07	19.93	5.00
Legs (g·kg cold carcass weight)	31.99 ^b^	32.88 ^ab^	33.93 ^a^	33.88 ^a^	0.42
Scapular fat (g·kg cold carcass weight)	0.84 ^a^	0.57 ^b^	0.55 ^b^	0.61 ^b^	0.05
Kidney fat (g·kg cold carcass weight)	1.99	1.59	1.69	2.34	0.02
^3^ Meat (g·100 g^−1^ of legs meat)	64.86 ^b^	69.71 ^ab^	68.71 ^ab^	73.26 ^a^	1.56
^3^ Bone (g·100 g^−11^ of legs meat)	33.10 ^a^	32.49 ^a^	29.38 ^a^	23.64 ^b^	1.37
^3^ Dissectible fat (g·100 g^−11^ of legs meat)	1.03	0.80	0.91	1.06	0.10

^1^ C = control, SY = 2.5% jackfruit seeds, PY = 2.5% jackfruit pulp, CY = 2.5% jackfruit peel. ^2^ SEM: Standard Error Media. ^3^ Variables were calculated with regard to leg weight. ^abc^: Different superscript lowercase letters among rows indicate statistical differences (*p* < 0.05).

**Table 5:** Least squares mean of quality meat from rabbits fed with different jackfruit (*Artocarpus heterophyllus* Lam.) sections.

Variable	Treatments ^1^				SME ^2^
	C	SY	PY	CY	
L*	57.26 ^ab^	56.92 ^b^	58.44 ^a^	55.42 ^c^	0.36
a*	4.19 ^b^	4.97 ^a^	4.85 ^a^	3.78 ^b^	0.16
b*	0.54	1.00	0.61	0.45	0.15
Chroma	4.52 ^b^	5.35 ^a^	5.16 ^a^	4.02 ^b^	0.16
Hue	17.97	17.21	17.36	15.57	1.32
pH	6.88 ^bc^	6.78 ^c^	6.94 ^ab^	7.09 ^a^	0.40
WHC ^3^ (%)	33.87 ^a^	26.82 ^b^	15.36 ^d^	19.60 ^c^	0.89

^1^ C = control, SY = 2.5% jackfruit seeds, PY = 2.5% jackfruit pulp, CY = 2.5% jackfruit peel. ^2^ SEM: Standard Error Media. ^3^ WHC = water-holding capacity. ^abcd^: Different superscript lowercase letters among rows indicate statistical differences (*p* < 0.05).

**Table 6:** Least squares mean of cooking losses and texture profile analysis of meat from rabbits fed with different jackfruit (*Artocarpus heterophyllus* Lam.) sections.

Variable		Treatments ^1^				SME ^2^
		C	SY	PY	CY	
Cooking loss		13.57 ^a^	11.78 ^b^	14.00 ^a^	13.80 ^a^	0.39
TPA ^3^	Hardness (N)	17.90 ^a^	16.83 ^a^	14.41 ^b^	10.47 ^c^	0.81
	Resilience	0.19	0.19	0.20	0.20	0.007
	Cohesiveness	0.63	0.64	0.61	0.62	0.01
	Springiness	0.64	0.62	0.60	0.61	0.01
	Chewiness (N)	8.94 ^a^	7.95 ^ab^	6.20 ^bc^	4.49 ^c^	0.84

^1^ C = control, SY = 2.5% jackfruit seeds, PY = 2.5% jackfruit pulp, CY = 2.5% jackfruit peel. ^2^ SEM = Standard Error Media. ^3^ TPA = texture profile analysis. ^abc^: Different superscript lowercase letters among rows indicate statistical differences (*p* < 0.05).

**Table 7:** Least squares mean for the sensory analysis of the meatballs made from rabbits fed with jackfruit (*Artocarpus heterophyllus* Lam.).

Variable	Treatments ^1^				SME ^2^
	C	SY	PY	CY	
Odor	4.38 ^a^	4.24 ^a^	4.05 ^ab^	3.40 ^b^	0.26
Hardness	3.94 ^ab^	3.99 ^a^	3.42 ^b^	3.79 ^ab^	0.21
Juiciness	5.17	5.24	5.15	5.43	0.24
Taste	6.25 ^b^	7.25 ^a^	6.61 ^ab^	6.85 ^ab^	0.25
General acceptability	6.61 ^b^	7.50 ^a^	7.28 ^a^	7.27 ^a^	0.21
Whiteness intensity ^3^	4.54 ^a^	3.76 ^b^	4.58 ^a^	2.14 ^c^	0.22
Redness intensity ^3^	3.88 ^c^	4.82 ^b^	4.61 ^b^	6.13 ^a^	0.22
Yellowness intensity ^3^	2.18 ^a^	1.69 ^ab^	2.14 ^ab^	1.41 ^b^	0.27

^1^ C = control, SY = 2.5% jackfruit seeds, PY = 2.5% jackfruit pulp, CY = 2.5% jackfruit peel. ^2^ SEM: Standard Error Media. ^3^ Raw meat. ^abc^: Different superscript lowercase letters among rows indicate statistical differences (*p* < 0.05).

## Data Availability

The datasets used and analyzed during the current study are available from the corresponding author on reasonable request.
